# A rather dry subject; investigating the study of arid-associated microbial communities

**DOI:** 10.1186/s40793-020-00367-6

**Published:** 2020-12-01

**Authors:** Peter Osborne, Lindsay J. Hall, Noga Kronfeld-Schor, David Thybert, Wilfried Haerty

**Affiliations:** 1 Earlham Institute, Norwich Research Park Innovation Centre, Colney Lane, Norwich, NR4 7UZ UK; 2 Gut Microbes & Health, Quadram Institute Bioscience, Norwich Research Park, Norwich, NR4 7UQ UK; 3 Chair of Intestinal Microbiome, School of Life Sciences, ZIEL – Institute for Food & Health, Technical University of Munich, 85354 Freising, Germany; 4 School of Zoology, Tel Aviv University, Tel Aviv-Yafo, Israel; 5 EMBL-EBI, Wellcome Genome Campus, Hinxton, Cambridgeshire CB10 1SD UK

## Abstract

Almost one third of Earth’s land surface is arid, with deserts alone covering more than 46 million square kilometres. Nearly 2.1 billion people inhabit deserts or drylands and these regions are also home to a great diversity of plant and animal species including many that are unique to them. Aridity is a multifaceted environmental stress combining a lack of water with limited food availability and typically extremes of temperature, impacting animal species across the planet from polar cold valleys, to Andean deserts and the Sahara. These harsh environments are also home to diverse microbial communities, demonstrating the ability of bacteria, fungi and archaea to settle and live in some of the toughest locations known. We now understand that these microbial ecosystems i.e. microbiotas, the sum total of microbial life across and within an environment, interact across both the environment, and the macroscopic organisms residing in these arid environments. Although multiple studies have explored these microbial communities in different arid environments, few studies have examined the microbiota of animals which are themselves arid-adapted. Here we aim to review the interactions between arid environments and the microbial communities which inhabit them, covering hot and cold deserts, the challenges these environments pose and some issues arising from limitations in the field. We also consider the work carried out on arid-adapted animal microbiotas, to investigate if any shared patterns or trends exist, whether between organisms or between the animals and the wider arid environment microbial communities. We determine if there are any patterns across studies potentially demonstrating a general impact of aridity on animal-associated microbiomes or benefits from aridity-adapted microbiomes for animals. In the context of increasing desertification and climate change it is important to understand the connections between the three pillars of microbiome, host genome and environment.

## Introduction

Water is essential for all known forms of life [1] and a stable arid environment is characterised by low annual precipitation (depending on location from 500- < 10 mm precipitation annually), with desertification occurring with greater loss than gain [2]. Approximately 30% [3] of the land surface area of the Earth is classified as ‘arid’. However even extreme deserts, including both hot and cold extremes and areas with exceptionally little precipitation, are still home to a wide diversity of life from microscopic [4, 5], to large mammals [6, 7] and long lived charismatic flora [8]. It is important therefore to understand how life is able to survive and adapt to the challenge of obtaining and retaining water, along with the other threats to animal life listed in Table 1. Aridity driven adaptations in plants [17, 18] as well as behavioural adaptations in animals [19] have already been well described and will not be addressed in this review. Research has demonstrated the importance of microbial organisms when living as members of associated communities on and in animals; for growth and development [20], dietary necessity [21] and for maintaining expected behaviour [22]. It is reasonable therefore to infer that the microbiota of animals resident in arid environments contributes to their host’s fitness in such harsh conditions. This may be through mechanisms already known from the study of microbiota in model organisms or by unique mechanisms.

**Table 1:** Examples of environmental factors which make arid environments inhospitable for animal life, and associated challenges faced by living organisms

Arid environmental factor	Challenges	Example animal adaptations
Lack of food	Extreme seasonality of food sources Requirement for multiple different food sources, or in contrast, to specialise to a single food source Travel longer distances to find food Increased exposure to predators Depressed metabolism	Switch lifestyle to nocturnal to access more different food sources - potentially also with greater water content [9]
Lack of water	Dehydration Reduced metabolic rate Reduced ability to manage body temperature Behavioural changes increase risk of predation i.e. sheltering to reduce water loss leading to exposure to predators	Reduce urine production and concentrate any produced [10] Store greater amounts of water in the body [11] Changes in activity patterns [12]
Extremes of temperature	Hyperthermia ● Protein denaturation ● Dehydration from increased panting or sweating ● Multiple organ failure Hypothermia ● Frostbite ● Water in body freezing ● Metabolic rate falling below survival baseline ● Loss of heat from extremities	Tolerate increased temperatures through seeking shelter from the heat of the sun [13] Tolerate freezing through production of specialised compounds and antifreeze proteins [14]
Extremes of salinity	Herbivores need ability to digest salty plant matter without losing excess water during digestion Need to maintain water balance in face of osmotic challenges from consuming salty water	Salt glands which can excrete salt from the body depending on dietary intake and internal osmotic balance [15]
Elevated UV-C and UV-B exposure	Increased risk of genetic damage from UV irradiation of external body surfaces	Increased skin, fur or carapace pigmentation [16]

Here we review the interactions between arid environments and the microbial communities which inhabit them along with the typical stresses these environments present. We specifically highlight and compare studies in arid-adapted animal microbiota, investigating patterns across studies, potentially demonstrating a general impact of aridity on animal-associated microbiomes or benefits from hosting aridity-adapted bacteria, fungi and archaea. We use the terms ‘desert’ and ‘arid’ interchangeably throughout the piece (as naturally occurring arid environments are referred to as deserts), typically naming the desert in question when relevant and indicating if a hot or cold desert is being discussed.

In this review we:

I. Review environmental microbiology studies in arid environments through the different environmental factors acting on bacteria, fungi and archaea
II. Describe animal physiological adaptations to aridity & highlight animal-associated microbiomes and their roles
III. Review arid-associated animal microbiomes, examining two camels in particular and the impact on microbiomes of environmental factors associated with aridity
IV. Discuss some of the challenges and opportunities around studying arid animal and environmental microbiomes

Increased global desertification [23–25], accelerating climate change [26] and changing land management [27] highlight the importance of better understanding arid ecosystems and animal adaptations to them. There have been comparatively few environmental microbiology studies on arid environments though in the hunt for extremophiles and pharmaceutically useful compounds there have been some focussed investigations of particular locations [28–31]. Animal-associated microbiota research has often been a component of a larger investigation into a particular host species [32] rather than a systemic approach considering the microbiome as a feature of animal life in arid environments.

## Aridity and environmental microbiology

Microorganisms can be found in almost all environments, adapting over millions of years to survive and thrive in conditions ranging from extremely hostile [33–35] to resource rich [36, 37]. Prior researchers were limited by the need to culture microorganisms, significantly impacting the scope of investigations on microbial diversity. Recent studies have indicated the importance of ‘culture-omics’ [38, 39] for creating large-scale microbial collections for mechanistic analysis and classifying metagenomic results which could not be assigned purely from bioinformatics. This has allowed investigation of environmental [40] and animal-associated [41] bacteria, fungi and archaea detected through bioinformatics and sequencing, but previously difficult to culture. The ability to sequence environmental samples and analyse genetic material without a need to grow microorganisms has allowed more accurate and large-scale accounting of existing diversity [42–44]. With the advent of new techniques and reduced sequencing cost, came a greater understanding of the influence of abiotic and biotic factors acting on microbial communities. Temperature [45–48], UV exposure [49, 50], salinity [51, 52], humidity [53], pH [54], irradiation [55], pressure [56], pollution [57] and oxygen concentration [58] have all been demonstrated to influence microbiomes.

A similar suite of challenges is encountered in almost all arid environments, foremost amongst these being the lack of free water. These environmental stresses may lead to the establishment of new species, affect the composition of a microbial community (presence or absence of given members) and the relative abundances of species in a community (the level at which members are present).

### Desiccation - lack of water

Lack of free water is the defining trait of arid environments, irrespective of temperature [59]. From a microbial perspective the lack of water presents the same dangers as those faced by macroscopic organisms along with uniquely microscopic challenges. In deserts where free water availability is low and the medium in which activity occurs is largely absent, Ho-Kyung Song *et. al.* [60] found that desiccation led to selection against motility associated proteins within their studied bacteria. They note that these proteins are associated with flagella; and this selective pressure may not be seen with alternative methods of motility. Multiple studies across different natural environments demonstrate reduced microbial diversity with desiccation [61–63] compared to sites with greater water availability. This may come from the impaired ability to obtain nutrients from free-moving water [64]. Desiccation can also lead to decreased production of anti-competition compounds. Fierer *et. al.* hypothesised that the significantly lower production of antimicrobial murein hydrolases, along with reduced abundance of antibiotic resistance genes are associated with the greater environmental pressure on prokaryotic and eukaryotic microorganisms over competition [65] as those compounds would require water for distribution. Le *et. al.* reported the production of potential osmoprotectants (such as osmoprotective proline) to be upregulated in the microbial communities of hypoliths from the Namib Desert and Antarctica [66]; similar adaptations were observed amongst bacteria living on dry city surfaces like metal and glass in New York [67]. Anderson *et. al.* found desiccation tolerance of an Archaeon increased when EPS (extracellular polysaccharide) production increased; additionally reporting increased tolerance of heat and oxygen stresses in desiccated versus control cells [68]. Due to environmental challenges, specific reproductive strategies may be employed. For instance, fungi living on extremely dry surfaces using meristematic development in order to reproduce without requiring water for dispersal [69, 70]. Within broader arid environments, relatively moist sites tend towards richer and larger microbial communities [71, 72], demonstrating the intensive selective pressure of aridity. It is worth noting that seemingly desiccated environments can in fact contain tiny water droplets home to bacteria, fungi and archaea surviving in otherwise deadly conditions [73], as well as dormant bacteria and fungi which revive and become metabolically active after an increase in moisture [74].

### Temperature - bake or freeze

Aridity and extremes of temperature are commonly found together. Antarctica and the Sahara are two clear examples of desiccation and dangerous temperatures making survival extremely difficult [75]. Multiple studies have investigated microorganisms surviving and thriving in locations with extreme temperatures [76–81]. In a hot environment, Armstrong *et. al.* found that the soil microbial communities of gravel plains in the Namib desert remained constant over time [82], speculating that this is likely due to the stable (though hostile) environmental conditions experienced throughout the majority of the study period. The stress of high temperature has been shown to lead to increased production of heat shock proteins [83]; high temperatures in geothermically active soils in Antarctica have also been shown to influence microbial community composition and may help explain the presence of thermophilic Archaea closely related to those found in similar hot environments thousands of miles away [84]. Cockell *et. al.* found that higher temperatures limited microbial diversity when other conditions were well-suited for life in and around Hawaiian fumaroles [85]. As expected, high temperatures have been shown to interact with the other stresses associated with aridity to influence microbial community composition - such as favouring endospore forming Firmicutes [86]. This supports the findings by Savage *et. al.* that elevated temperatures still allowed more diverse microbial communities than those possible when temperatures were combined with other abiotic stresses [87]. In cold environments, production of cold shock proteins has been documented [88]. Antarctic bacteria are known to produce antifreeze proteins [89], Liljeqvist *et. al.* found a gene predicted to code for production of an antifreeze protein in their metagenomic study of an acid mine drainage stream in northern Sweden [90]. Adaptations to cold temperatures have also been noted in fungi through increased production of unsaturated lipids in the cell membrane [91], maintaining membrane fluidity. Cryotolerant fungi may also accumulate cryoprotectants like glycerol [92].

### Radiation

Drastically reduced coverage from clouds or vegetation in arid environments means exposure to damaging UV light [93, 94]. UV exposure levels have been shown to influence microbial community composition [95]. This includes dry environments where desiccation can lead to greater difficulties in tolerating UV irradiation [96]; though some other studies disagree [97]. Adaptation for UV exposure is typically through pigmentation. As such, environmental sampling of UV irradiated sites in Antarctica [98] and Tibet [99] identified increased pigmentation in UV irradiation tolerant *Hymenobacter*. A number of studies looking at the microbial residents of solar panels [100, 101]; including panels in Antarctica, the Arctic and the Mediterranean all found *Hymenobacter* as the most abundant genus [102]. Greater numbers of genes associated with antioxidant production and DNA repair have been noted in Archaea and Bacteria in heavily UV exposed environments [103–105]. These observations raise the possibility that extremely effective DNA repair mechanisms may also be a means of adapting to aridity. Pacelli *et. al.* subjected desiccation resistant Antarctic fungi to gamma radiation levels much greater than any found in nature and speculate that observed tolerance may use known DNA repair mechanisms associated with UV irradiation and dehydration [106]. Investigation by Selbmann *et. al.* in Antarctic fungi resistant to UV-B exposure led them to suggest that thick and highly melanised cell walls rather than enhanced DNA repair systems were the principal factor in tolerance of UV irradiation [107]. Jones and Baxter review in depth some methods of tolerating UV stress in Archaea which may be translatable into work with other microorganisms [108].

### Salinity

Some arid and semi-arid environments are heavily subjected to salt stress [109]. Intertidal zones and beaches experience either daily coverage with seawater or large quantities of salt deposition from wind off the ocean. Typically, the adaptations employed to protect microorganisms against desiccation also confer protection against salinity - the loss of water being a shared peril. Salinity in arid or other extreme environments can act as an independent factor controlling community composition; with microorganisms needing to be halotolerant in addition to being capable of dealing with other stresses. Management of osmotic potential in a saline environment is a long-term issue and so may require greater dedication of resources than acute salt stress caused by decreasing volumes of water; or employ particular pathways. This was noted by Molina-Menor, *et. al.* when examining the microbial community of rocks in the intertidal zone of the Mediterranean [110], and distinct microbiomes from more saline areas of larger environments have been recorded [111]. Production of hydrophobins [112] and accumulation of salt-stress specific solutes [113] were observed in fungi from saline environments.

### From environmental to animal microbiomes

Comparisons of animal microbiomes to those of their surroundings within extreme environments, such as hot and cold deserts, are currently few in number and not covered in detail in this review. The external conditions of animals in arid environments are liable to be similar to their wider environment. Although behavioural adaptation will alleviate some of the effects of environmental stresses, members of the skin microbiota will be subject to similar stresses as other environmental communities. Salt levels in the intestinal tract of the hot-desert dwelling Fat Sand rat (*Psammomys obesus*) are similar -at least initially- to those of the saltbush they consume [114], and water content in the faces of desert species is very low. Internal conditions in an animal will differ from the external environment, but potentially not as greatly as between an arid and a wet environment. Therefore arid-adapted animals may host extreme points on different gradients within the larger arid environment, in terms of water availability, temperature (for ectotherms) or salinity; whilst being within the standard range for other environmental variables (e.g. temperature on and within an ectothermic cold desert insect).

### Animal adaptation to aridity & animal-associated microbiomes

The same factors (e.g. lack of water, extremes of temperature, restricted energy sources) associated with arid environments influence both microbial and animal communities. In order to survive and thrive within such habitats, animals have developed a suite of physiological and behavioural adaptations. These reduce energy expenditure and water loss [115], use microclimates or seek increased water intake from other sources [116–118]. Table 1 provides some examples of animal adaptations in the face of specific stresses; to mirror the focus on mammals in metagenomic studies we focus on non-bird vertebrate adaptations to aridity. A number of animal species restrict their urine production in response to acute water stress, from Merino sheep [119] to ostriches [120] and some toads [121]. These species demonstrate the ability to reduce and concentrate [122–124] the amount of urine they produce through specific renal adaptations [125] such as the elongation and enlargement of the renal papilla [10, 126, 127], as well as changes to the distribution of aquaporin proteins [128]. Additionally, animals can store water for use over a longer period, as dromedaries do with their forestomach [129]. Some animals may take advantage of abnormal water sources to survive in arid locations [130], including non-xeric animals [131]. A common behavioural adaptation is to seek shelter, shade or microclimates in hot arid environments to limit heat stress and exposure to dry air; thus reducing evaporative water loss [132]. Switching to a nocturnal rather than diurnal activity pattern is another means of reducing water stress in hot arid environments [133] and of adapting to increasing aridity with climate change [9]. Adaptation for reduced basal metabolic rates in birds along an aridity gradient was uncovered by Tieleman *et. al* [134]. Low basal energy demands (basal metabolism) are common in hot desert endothermic animals, reducing both the need to forage in hot desert conditions and lung ventilation thereby reducing evaporative water loss [134, 135]. Many endotherms reduce metabolic rate and water loss further through torpor, a state which can last from hours to months and is a reduction of body temperature and other physiological processes [118, 136–138]. These changes can be programmed, timed processes or direct responses to environmental conditions [118]. Larger body size may also confer some protection against desiccation, as seen in *Anopheles gambiae* [139] and camels [140, 141]. Invertebrate survival of extreme dehydration and temperatures is reviewed by Watanabe [142] and Somme [143], showing a suite of morphological, behavioural and physiological changes across a range of species.

While all these studies focused on an organism’s adaptation to aridity, it is important to consider the influence of the microbiome, often called the ‘second genome’, on arid-adapted animals as a potential contributor to aridity tolerance. This is a dynamic relationship, in which the host animals’ adaptations for aridity will directly influence the different niches it provides for potential microbial colonisation and the microbiome may negate the need for host genomic adaptation to aridity. The development of new sequencing methods and increased computational power, coupled with innovative software and analysis approaches have made large scale microbiome investigations more accessible [144, 145], highlighting the vital role they play in host development and health [146]. Research has often been directed to the intestinal microbiomes of ruminants [147–153] and other commercially valuable species [154–156]. By virtue of ease of access, other studies have tended to be on domesticated species [157], or wild species which can be more conveniently reached and investigated [158].

Broadly, arid animals have not been the subject of as much metagenomic research as those found in more hospitable environments. This has led to focus on a small number of species, and less connection across the field between environmental, host and metagenomic factors in arid animals.

## Arid animal microbiomes

Beyond environmental microbiomes, and aside from plant-associated microbiomes which are outside the scope of this review (see [159, 160]), the other potential location for microbial communities in arid environments is in association with animal hosts. Here we discuss animal microbiomes influenced by some of the aridity associated factors discussed above, then explore in greater detail Camels and Muskoxen, which have received more investigation than most in this area.

### Incidental aridity - animal microbiomes influenced by abiotic factors shared with arid environments

Before moving on to some specific arid-adapted animals it is useful to look at animal microbiomes which may have been influenced by aridity directly or indirectly - or by factors also found in arid environments, similar to the environmental microbiomes discussed above. The comparatively reduced levels of water available in arid environments, whether as humidity, surface moisture or precipitation, limits the ability of macroscopic life to develop [161, 162]. From the perspective of animal host organisms this leads to a tendency for specific diets, becoming more limited as aridity increases and diversity of plant and animal life falls [163, 164]; this has been reported in numerous organisms [165–168]. The human skin microbiome has distinct correlations between moisture levels and community composition [169, 170]; this could offer some comparative references if skin-microbiome studies of arid-adapted animals are conducted. Diet plays a large, potentially dominant role in establishing the intestinal microbiota of animals [171] and can influence the microbiota of other body areas as well [172, 173].

Limited food and water availability due to arid conditions can lead to concentration of animals in sites with accessible water or a shared source of nutrition - this consumption from the same site and possible close quarters may help explain the proximity based correlation in microbiota composition (likely through range overlap leading to similar microbial exposure) noted by Couch *et. al.* in Mojave desert-dwelling Bighorn sheep [174] faecal microbiota. Other stresses found in arid environments have been examined in terms of animal-associated microbiomes, both experimentally and in observation of natural conditions. Direct links have been observed between temperature and animal-associated microbiomes in Humpback whales (2 °C to − 2 °C) [175], Fruit Flies (13 °C versus 31 °C) [176], Silkworms (transient exposure to 37 °C after rearing at 25 °C) [177] and Tilapia (24 °C versus 12 °C) [178]. This influence can be profound or act in more subtle ways, as Li *et. al.* noted in *Xenopus tropicalis* whereby decreasing temperature altered beta but not alpha-diversity of the gut microbiome [179]. Interestingly, some of these temperature dependent changes in host microbiomes reflect either changes in the host temperature (the fruit flies and silkworms for instance) or in the environmental temperature (Humpback whales). In some instances, these studies have found that the microbiomes subject to influence by heat stress also impact their hosts; Fontaine *et. al.* finding that temperature induced changes in salamander intestinal microbiota influenced energy uptake from digesting food [180]. Givens found that a change in water temperature surrounding some *Fundulus hetereoclitus* (Mummichog) corresponded with a change in relative abundance of different species of *Vibrio* in the intestinal microbiota which may have been connected with increased mortality [181].

Sullam *et. al.* reported that different levels of salinity influenced the gut microbiota of fish - and that these saltwater fish intestinal communities bore similarities to environmental samples from saltwater [182]. Investigation of Atlantic Salmon found a less diverse intestinal microbiota in those acclimated to saltwater than those living in freshwater [183]. 16S investigation of the exposed facial skin of the Black and Turkey vultures by Mendoza *et. al.* identified *Psychrobacter cryohalolentis* and *Psychrobacter arcticus*, which whilst commonly found in cold environments are known to be halotolerant [184] potentially explaining their presence on the warm but saline surface. Given similarities in environmental microbiomes between saline and arid environments, it is possible that dehydration might influence animal microbiomes in a similar way to salt stress; favouring the same functions and potentially related taxonomic compositions. These studies examine environmental salinity rather than that of internal fluids of the host, whether in the intestinal tract or elsewhere; it could be of interest to assess salinity within the host and potential impacts on the microbiota without an external change in salt levels; tied to consumption of a salt rich diet for instance.

Direct exposure to UV irradiation of animal microbiomes is restricted to those on the external surfaces of the body, skin [185], fur [186], scale [187], etc. Ghaly *et. al.* found that increased UV irradiation of mouse skin changed the intestinal microbiota - with changes detected at the phylum and genus level which may correspond with increased inflammation [188]. Investigation of New World vultures by Graves *et. al.* found that extremophiles tolerant of UV irradiation (and desiccation) accounted for the most abundant and third most abundant genera resident on pigmented plumage in a number of studied species [189]; *Hymenobacter* was the third most abundant genus, and as discussed above, also the most abundant on polar and Mediterranean solar panels as well as UV-irradiated environments in Tibet and Antarctica. Examination of the external surfaces of animals exposed to elevated UV levels, whether through altitude or relative immobility, may also find similarities between these communities and those of environments subject to significant UV irradiation.

### Camels and muskoxen, hot and cold arid environments

The single-humped Dromedary (*Camelus dromedarius*), the double-humped domesticated Bactrian (*Camelus bactrianus*) and wild Bactrian (*Camelus ferus*) camels are animals found in hot or cold arid environments, from the Australian Great Sandy Desert [190] to the steppes north of the Gobi Desert [191]. They are excellent cases for comparison of the influences of aridity on animal microbiomes. As with the majority of animal-microbiota studies the focus of the camel investigations has been the intestinal microbiota, ranging from covering multiple sites along the gastrointestinal tract to only sequencing faecal samples. Muskoxen, *Ovibos moschatus*, are ruminants found in the wild roaming the High Arctic of Greenland, Canada and Alaska; as well as being reared commercially elsewhere in the High Arctic [192–194]. They have been investigated as one of many ruminant species to have had their intestinal microbiomes examined, and are of interest in the context of this review as they inhabit a cold arid environment.

Gharaechahi *et. al.* used 16S rRNA sequencing to study the microbiota of solid and liquid fractions of three female Dromedaries, finding evidence that the core microbial community was highly conserved between the individuals [195], which may be a consequence of limited diets. Interestingly, aridity limiting the diversity of potential energy sources in the diet may explain increased microbial community diversity and richness in Muskox at more northern latitudes as found by Bird *et. al.* [196]; potentially preventing a small number of bacteria or archaea specialising in an abundant energy source from dominating the community. He *et. al.* used 16S rRNA sequencing to investigate the microbiota composition along the digestive tract of the Bactrian camel and found a large number of unclassified Ruminococcaceae in the ileum and large intestine; which they suggest might enable survival on salt-tolerant, difficult to digest forage in their arid habitat [197]. An early investigation, utilising metatranscriptomics rather than metagenomics and focusing on rumen eukaryotic residents in the Muskox found enrichment for CAZy gene families [194]; also found to be enriched in the Dromedary intestinal microbiome by Gharechahi & Salekdeh though from prokaryotic sources [198]. This suggests that investigation of the metatranscriptome of the Dromedary may yield similar results - though the contribution of eukaryotic and prokaryotic members of the microbiota may differ. Other work by He *et. al.* on the Bactrian intestinal microbiota, employing 16S rRNA methods, found increasing complexity and stability of the community with age [199]. They also observed that some seasonal variation of the forestomach microbiota may occur [193]. Shotgun metagenomic sequencing by Gharechahi & Salekdeh to investigate the intestinal microbiome of Dromedaries [198] found a similar pattern for relative abundances of phyla previously identified by Gharechahi *et. al.* highlighting the difference in relative abundance of Verrucomicrobia detected between Bactrian and Dromedary camels. This may be a consequence of different temperature stresses on the host, different diets or potentially the relative abundance of Verrucomicrobia in the soils of their respective habitats.

These results suggest a general trend in camel species for intestinal microbiota which provide maximum resource extraction from the harsh environment in which they live, with limited and typically static diets - a mutually beneficial arrangement developing between the camels and these microorganisms. It is interesting to note the similarities across these studies, along with the older Dromedary forestomach microbial investigation by Bhatt *et. al.* [200] as regards the most abundant phyla detected. Salgado-Flores *et. al.* published the first metagenomic study of the Muskoxen rumen in 2016, utilising 16S rRNA techniques [201]. Bacteroidetes and Firmicutes were the dominant phyla, as with many other sequenced intestinal microbiomes, however they did note that 53.7–59.3% of bacterial sequences couldn’t be characterised. Also, the ratio between relative abundance of Firmicutes and Bacteroidetes was greater as compared to other ruminant intestinal microbiomes, 70.7–81.1%: 16.8–25.3%. This may be an indication that the environment, diet or a combination of the two in their arid habitat favours members of Firmicutes more generally than Bacteroidetes. However, reporting the most abundant phyla is not necessarily the most useful finding to explore given that research has found the same phyla predominant in the intestinal microbiota of humans [165], pigs [155], baleen whales [166] and in fact (at different relative positions) in the soil of the Atacama Desert [202]. In their later work Gharechahi & Salekdeh used shotgun sequencing methods to investigate functional traits of the camel intestinal microbiome. The authors determined that despite taxonomic similarities to a number of published rumen intestinal microbiomes it was functionally distinct from them and more akin to the Moose rumen microbiome in certain regards [198]. Table 2. shows the Bactrian and Dromedary camel metagenomic studies cited in this review including potential links between relatively abundant or notable taxa and their roles. Both Camel species and Muskox share some notable trends, with members of *Lachnospiraceae* and *Prevotellaceae* taking up a sizeable proportion of classified bacterial reads. This is likely to be due to similarities in diet courtesy of similar traits in plants needed to survive in both cold and hot arid environments.

**Table 2:** Summarises metagenomic investigations of camel species including the host species, sequencing technology used, notable prokaryotic taxa detected and postulated roles in the microbiome for the listed taxa. Unless otherwise indicated Dromedary camels were sampled from hot environments, Bactrian from cold; for ^a^ indicated studies where no clear location is given for location animals were sampled from

Sequencing method	No. sampled organisms	Host organism	Prokaryotic taxa of interest	Potential role of prokaryote(s) in microbiome	Study
16S rRNA	3	Dromedary^a^	*Prevotella ruminicola*	Produce glycoside hydrolyse enzymes Synergise with fibrolytic bacteria to improve fibre digestion	Gharechahi J. *et. al.* 2015 [195]
			*Ruminococcus flavefaciens*	High efficiency in degrading crystalline and amorphous cellulose	
			*Fibrobacter succinogenes*	Prolific cellulose degrader	
16S rRNA	18	Bactrian	*Blautia* species	May provide anti-inflammatory effects in young camels	He J. *et. al.* 2019 [199]
			*Christensenellaceae* members	May help regulate intestinal environment Linked to immunomodulation and healthy homeostasis	
16S rRNA	11	Bactrian	Unclassified Ruminococcaceae	May contribute to further feed fermentation to cope with low quality forage	He J. *et. al.* 2018 [197]
			Unclassified Clostridiales		
			*Akkermansia* species	May help prevent diabetes even with high blood glucose levels	
				May help prevent hypertension even with diet high in salt	
Whole genome shotgun	3	Dromedary^a^	*Fibrobacter succinogenes*	Potential lignocellulose degrader	Gharechahi J. *et. al.* 2018 [198]
			Member of *Ruminococcus*	Degrading crystalline cellulose	
			Members of Fibrobacteres	Help deal with diet rich in lignocellulose	
			Members of Spirochaetes		
			Members of Bacteroidetes	Utilise PUL enzymes to assimilate complex dietary carbohydrates	
Whole genome shotgun	6	Dromedary	*Bacteroides thetaiotaomicron*	Production of starch degrading enzymes	Bhatt V.D. *et. al.* 2013 [200]
16S rRNA	3	Muskox	Members of *Ruminococcaceae*	Help digest highly lignified winter forage diet	Salgado-Flores A [201].

## Considerations when studying arid microbiomes

The majority of studies discussed employ 16S rRNA sequencing methods, although a few have also utilised shotgun metagenomics. Many authors have used 16S rRNA approaches due to cost and availability of accessible tools. However 16S rRNA only allows genus level resolution and does not give an indication of functional potential. This is a particular issue when studying highly adapted hosts or environments where it is expected that evolutionary adaptation is at (or predicted to be at) the species/strain and functional level, rather than at genus level or higher. This reflects a trend in which many arid environments or arid-adapted animal microbiomes are sequenced with 16S rRNA and these results published with the caveat that any functional data they present is by necessity derived from the taxonomies they have generated. Frequent use is made of QIIME [203], mothur [204] and PICRUSt [205] - along with 16S rRNA databases such as Greengenes [206, 207] and Silva [208] to highlight what might be expected to be present in functional terms in the microbiome. Though 16S rRNA sequencing currently has advantages in terms of cost and (comparative) ease of use, the limitation to taxonomy-derived functional predictions can be an issue if functional diversity differs significantly from taxonomic measures. Shotgun sequencing enables the direct assessment and investigation of functional diversity within the microbial communities of arid environments, plants and animals.

Deeper sequencing power can create issues when studying novel hosts, as there may be a high proportion of novel microbial taxa present i.e. a high proportion of unidentified reads. This reflects the relative focus of animal-associated metagenomic studies on humans [209], mice [210] and others used as models for medical research [211]. Unless attempting de novo classification methods, taxonomy in metagenomics depends on databases of known (or likely) classifications against which reads from samples can be compared [212]. These are populated by researchers engaging in metagenomic and microbiological studies, thus trend towards easily cultivated or human-associated; though published datasets can be investigated for new genomes [213].

In their latter work, Gharechahi *et. al.* [198] compared some of the results of their whole genome shotgun sequencing of the camel rumen microbiome to results from their 16S rRNA investigation [195]. Of note are the differences in ability to resolve taxa to the species level, with 21% of the sequences in the metagenomic study being unclassifiable at the species level compared to 51.9% in the 16S rRNA investigation. It is possible that in the intervening years between the studies the growth in size and depth of taxa covered by databases may explain the difference. Both studies found less than 1% of the reads to be classified as Archaea, different methods finding similar results suggesting that Archaea are, proportionally at least, minor contributors to the Dromedary intestinal microbiome. They also speculate as to the differences in abundance of particular phyla depending on the different methodologies employed, which they say may be related to PCR bias. Interestingly for comparison of methods within the same species for metagenomic research, their use of 16S rRNA sequences extracted from metagenomically-assembled-genomes allows for an insight into how more populous and diverse databases can help metagenomic studies.

Work published in recent years highlights the importance in conservation of the microbiomes of animals moving into and out of captivity [214]. Conservation efforts might present an opportunity for shotgun sequencing microbiomes of arid-adapted animals. This would also help expand taxonomic and functional databases employed in metagenomic research. Some of the studies discussed above sampled from multiple locations within the intestinal tract, noting differences between them as well as with the faecal samples they obtained. Similar findings from other species including pigs [215], bats [216] and humans [217] suggest that where possible (and ethically sound) taking samples from internal body sites is necessary to fully understand arid-adapted animal microbiomes. Meng *et. al.* investigated intestinal microbiota of hot desert-dwelling weevils feeding on plant roots underground, finding that all of the 66 core weevil OTUs could be detected in the soil around the weevils; albeit at lower abundances [218]. This suggests that environmental sampling around any animals investigated might help us understand the origins and development of the microbiome. Where feasible it would be beneficial to obtain samples of the environmental microbiome to see what, if any relationship it has with the animal-associated microbial community. This would allow us to distinguish between transient microorganisms in the organism, environmental contamination or true residents. Ideally comparison across closely related species should be employed, as by Campbell *et. al.* (though not with arid-adapted organisms) [219], which will allow for better understanding of the interactions between host genome, environment and microbiome in studies of adaptation.

### Perspective and conclusion

Instead of lifeless wastes, arid environments are home to organisms from microscopic to enormous [220, 221]. Their living conditions are harsh, but still life is able to survive and thrive. Though diversity drops off as their home becomes more hostile, organisms have been discovered in the depths of Antarctica [222], the Atacama [28] and even in artificially desiccated environments [223]. Adaptations to aridity have been noted in environmental microbiomes as conferring survival advantages against other stresses which co-occur in those environments. As such it is likely that similarities exist between arid microbiomes and those found in hyper-saline, extremely cold, UV irradiated and hot microbiomes; though as moisture levels increase these similarities likely diminish. Within this context it is worth considering the extent to which animals in arid environments provide more hospitable refuges for environmental microorganisms, if potentially they allow for organisms present in small fractions externally to colonise, be fruitful and multiply. Future investigations comparing arid-animal microbiomes to those of their surroundings will need to take account of the impact of faeces and other excretions from the animals which may alter the microbial community in the environment around them [224]. Going forward it is worth considering whether seemingly divergent environmental conditions may in fact contain a similar stress which could impact microbial communities. When investigating environmental microbiomes it could be of benefit (if possible) to take detailed measurements of abiotic stressors and assess whether these may be directly - or through interactions with each other - responsible for community compositions. This may lead to discoveries of shared taxonomic or functional trends from distant and apparently dissimilar locations. It may be useful in addition to sample harsh environments which are not the most extreme, sampling a range of warm pools rather than the hottest spring water for instance; or taking transects across a harsh environment as opposed to multiple samplings from the saltiest or most irradiated sites.

If conducting research on arid animal microbiomes in the future it may be helpful to take environmental samples from sites where the animals feed, rest and otherwise spend their time. This could allow the determination of the extent to which composition of animal-associated microbiomes is purely a consequence of allowing minor members of the wider environmental microbiome to thrive. It may also be interesting to observe in captive animals if differences in diet and microbiome come from the altered diet they consume in captivity or from the different environmental microbiome in which they and their food are kept. Both traditional and novel methods of culturing will have a place in future studies of arid animal and environmental microbiomes, as harsh conditions can be more easily and accurately replicated in the laboratory; enabling the conversion of detected genotypes into observable phenotypes. Functional understanding of arid animal-associated microbiomes may require the use of in vivo models to be completely elucidated; possibly leading to findings which can be translated into industrial applications. In their recent discussion of the ‘Eco-holobiont’ Singh *et. al.* highlight how environmental factors, environmental microbiomes, animal genomes and animal-associated microbiota may interact to paint a complete picture of the micro-macroscopic relationships shaping the world [225]. Ribeiro *et. al.* demonstrate that metagenomic investigation can be a crucial component of well-rounded research into the life and adaptation of an arid-adapted animal, along with environmental, metabolic and genomic investigations [32]. Arid environments could offer a very useful proof of concept for this philosophical approach, with clear environmental influences and comparatively simple living communities. As climate change threatens arid environments and their inhabitants around the globe it is increasingly important that we fully understand the functional and taxonomic composition of the microbial communities they host so we can best protect and harness them [226].

## Data Availability

Data sharing not applicable to this article as no datasets were generated or analysed during the current study.

## Abbreviations

UV: Ultra Violet Light
DNA: Deoxyribonucleic Acid
CAZy: Carbohydrate-Active enZYmes
QIIME: Quantitative Insights Into Microbial Ecology
PICRUSt: Phylogenetic Investigation of Communities by Reconstruction of Unobserved States
PCR: Polymerase Chain Reaction
OTU: Operational Taxonomic Unit
